# Outbreak of Fatal Childhood Lead Poisoning Related to Artisanal Gold Mining in Northwestern Nigeria, 2010

**DOI:** 10.1289/ehp.1103965

**Published:** 2011-12-20

**Authors:** Carrie A. Dooyema, Antonio Neri, Yi-Chun Lo, James Durant, Paul I. Dargan, Todd Swarthout, Oladayo Biya, Saheed O. Gidado, Suleiman Haladu, Nasir Sani-Gwarzo, Patrick M. Nguku, Henry Akpan, Sa’ad Idris, Abdullahi M. Bashir, Mary Jean Brown

**Affiliations:** 1 Healthy Homes and Lead Poisoning Prevention Branch, National Center for Environmental Health, Centers for Disease Control and Prevention, Atlanta, Georgia, USA; 2 Epidemic Intelligence Service, Centers for Disease Control and Prevention, Atlanta, Georgia, USA; 3 Agency for Toxic Substances and Disease Registry, Atlanta, Georgia, USA; 4 Guy’s and St Thomas’ NHS Foundation Trust and King’s Health Partners, London, United Kingdom; 5 Médecins Sans Frontières, Amsterdam, the Netherlands; 6 Nigerian Field Epidemiology and Laboratory Training Program, Abuja, Nigeria; 7 Centers for Disease Control and Prevention, Abuja, Nigeria; 8 Nigerian Federal Ministry of Health, Abuja, Nigeria; 9 Zamfara State Ministry of Health, Gusau, Nigeria

**Keywords:** artisanal gold mining, childhood, environmental health, lead poisoning, nervous system

## Abstract

Background: In May 2010, a team of national and international organizations was assembled to investigate children’s deaths due to lead poisoning in villages in northwestern Nigeria.

Objectives: Our goal was to determine the cause of the childhood lead poisoning outbreak, investigate risk factors for child mortality, and identify children < 5 years of age in need of emergency chelation therapy for lead poisoning.

Methods: We administered a cross-sectional, door-to-door questionnaire in two affected villages, collected blood from children 2–59 months of age, and obtained soil samples from family compounds. Descriptive and bivariate analyses were performed with survey, blood lead, and environmental data. Multivariate logistic regression techniques were used to determine risk factors for childhood mortality.

Results: We surveyed 119 family compounds. Of 463 children < 5 years of age, 118 (25%) had died in the previous year. We tested 59% (204/345) of children < 5 years of age, and all were lead poisoned (≥ 10 µg/dL); 97% (198/204) of children had blood lead levels (BLLs) ≥ 45 µg/dL, the threshold for initiating chelation therapy. Gold ore was processed inside two-thirds of the family compounds surveyed. In multivariate modeling, significant risk factors for death in the previous year from suspected lead poisoning included the age of the child, the mother’s work at ore-processing activities, community well as primary water source, and the soil lead concentration in the compound.

Conclusion: The high levels of environmental contamination, percentage of children < 5 years of age with elevated BLLs (97%, > 45 µg/dL), and incidence of convulsions among children before death (82%) suggest that most of the recent childhood deaths in the two surveyed villages were caused by acute lead poisoning from gold ore–processing activities. Control measures included environmental remediation, chelation therapy, public health education, and control of mining activities.

Childhood lead exposure results in lower intelligence and behavior problems and negatively affects multiple body systems (Bellinger 2004; Canfield et al. 2003; Lanphear et al. 2005; Mendelsohn et al. 1998; Tellez-Rojo et al. 2006). Young children are particularly susceptible to lead exposure because of behavioral factors, such as frequent hand-to-mouth activities, and biological factors including greater gastrointestinal absorption and developing neurological systems (Bellinger 2004; Henretig 2006; Lidsky and Schneider 2003). Encephalopathy typically occurs with blood lead levels (BLLs) ≥ 100 µg/dL but can occur with BLLs as low as 70 µg/dL [Centers for Disease Control and Prevention (CDC) 2002; Henretig 2006]. Symptoms of acute lead encephalopathy include vomiting, changes in behavior, ataxia, convulsions, and coma (CDC 2002; Henretig 2006). Currently, childhood lead poisoning resulting in encephalopathy and death in developed countries is rarely reported, and the last documented child fatality from lead poisoning in the United States was in 2006 (Berg et al. 2006). However, hundreds of U.S. pediatric deaths from acute lead encephalopathy were recorded in the first half of the 20th century. For example, 202 deaths from childhood lead poisoning were recorded in U.S. cities from 1931 and 1940, with 25% occurring in Baltimore, Maryland (McDonald and Kaplan 1942). Most lead poisoning in this period occurred in urban settings from deteriorating lead house paint and lead-painted cribs. Lead-poisoned children in this era frequently had high BLLs. One study evaluating lead poisoning in 293 children from 1931 to 1970 found the mean BLL for children with mild, severe, and fatal acute lead encephalopathy to be 328 µg/dL, 336 µg/dL, and 327 µg/dL, respectively (National Research Council 1972).

Sources of global lead poisoning include lead mining and smelting, battery recycling, leaded gasoline, paint, traditional medicines, toys, and discarded electronic devices (Meyer et al. 2008). Lead poisoning is of specific concern for children in developing countries (Falk 2003). The World Health Organization (WHO) estimates that lead poisoning causes 0.6% of the global burden of disease and contributes to approximately 600,000 cases of intellectual disability in children annually (WHO 2010). During the past 20 years, moderate to high elevations of BLLs have been documented globally in clusters of children living in mining and smelting communities or areas where lead batteries are reclaimed (Brown et al. 2010; Garcia Vargas et al. 2001; Kaul et al. 1999; Lalor et al. 2006). However, only one report found in recent literature documents fatalities from childhood lead poisoning; in 2008 in Senegal, informal battery recycling was determined to be the likely cause of death of 18 children (Haefliger et al. 2009).

*Description of the outbreak.* During meningitis surveillance activities conducted February–April 2010, Médecins Sans Frontières (MSF) and local Nigerian public health officials observed higher than expected numbers of childhood illnesses and deaths in four villages in rural northwestern Nigeria. Most of the illnesses and deaths occurred in children < 5 years of age. Symptoms included vomiting, abdominal pain, headache, and convulsions. Ill children failed to respond to malaria treatment and empiric antibiotics. MSF and State Ministry of Health staff noted that all four villages participated in artisanal gold ore–processing activities, and heavy-metal poisoning was considered as a potential source of the illnesses. Venous blood samples, analyzed in Europe, from eight symptomatic children from an affected village indicated BLLs between 168 and 370 µg/dL. These concentrations exceed the U.S. CDC level of concern of 10 µg/dL and are well known to be fatal (CDC 2002). MSF notified Nigerian authorities of the results, and a team of international experts was mobilized to assist in the investigation and response to this outbreak.

In this report we describe the response and investigation of an outbreak of childhood lead poisoning with child mortality unprecedented in modern times. On 8 May 2010, the Nigerian Federal Ministry of Health assembled representatives from federal, state, and international organizations, including the Nigeria Field Epidemiology and Laboratory Training Program, CDC, and WHO, to join in ongoing MSF efforts to investigate and respond to the outbreak. The purpose of this emergency response was to determine the cause and contributing factors to the outbreak and identify and prioritize children < 5 years of age with lead poisoning for emergency chelation therapy. The urgent nature of the response and the limited resources necessitated quick investigation and intervention to stop the increasing rate of child fatalities in these villages. Although some adults in these villages were reported to have symptoms of lead poisoning, initial efforts focused almost entirely on children < 5 years of age. Young children are most susceptible to lead poisoning, and most of the fatalities in MSF clinics were among children < 5 years of age. Investigators hypothesized that the outbreak of lead poisoning was associated with artisanal gold ore–processing activities. Investigators used a three-pronged approach to investigate the outbreak that included a door-to-door survey, blood lead sampling, and environmental testing.

## Methods

*Cross-sectional survey.* Stakeholders, including Nigerian state health officials and MSF, recommended the two most affected villages (A and B) for participation. The investigation protocol was reviewed and approved by both the Nigerian government and the CDC. The investigation was conducted in accordance with the Declaration of Helsinki developed by the World Medical Association (2008).

After obtaining consent of village leaders, investigators conducted a cross-sectional, door-to-door survey from 23 May to 4 June 2010 to interview parents, sample blood from children < 5 years of age, and collect soil from households. Informed consent to administer the survey, draw blood, and collect environmental samples was obtained from the head of the household before survey initiation. Local health professionals and translators were trained in a 4-hr session on survey administration, venous blood drawing, and environmental sampling.

Each village consisted of numerous family dwellings separated by low walls. Investigators defined a compound as several multigenerational and multifamily dwellings enclosed by a common wall. All compounds in village A were eligible to participate in the survey. Because of time and logistical constraints, only compounds in the central area of village B were eligible to participate. The central area of village B included most of the compounds and common gathering places such as mosques, markets, and the residence of the head of the village.

The survey collected information about children < 5 years of age in each compound, including the number living in the compound, the number with a history of convulsions, the number who had died in the preceding 12 months, and their approximate date of death.

Before the investigation, the team heard that artisanal gold ore–processing activities were occurring in both villages. These ore-processing activities included *a*) breaking rocks into small gravel-size pieces (breaking); *b*) grinding rocks into a fine powder with a flour mill or mortar and pestle (grinding); *c*) washing ground ore powder with water to separate gold particles (washing); *d*) drying ground ore after washing (drying); *e*) using liquid mercury to amalgamate gold flakes (separating); and *f*) using heat to vaporize mercury from the gold mixture after amalgamation (melting). Both men and women in the villages participated in ore processing. Men typically processed ore in central locations around the village, and women processed ore inside family compounds. The survey included questions about gold ore–processing activities inside and outside the compound, household member and maternal processing activities, history of animal deaths within the compound, and the primary water source of the family. Before data collection, investigators mapped each village and marked the location of each family compound. Global positioning system (GPS) coordinates were taken at the entrance of each compound to facilitate compound identification and follow-up on blood and environmental testing results. At the end of the investigation, MSF staff visited the compounds and provided parents with blood lead test results of their children as well as information on chelation therapy and the medical management of lead poisoning.

*Blood sampling.* Venous blood was collected from children 2 months to 5 years of age. Phlebotomists attempted to draw blood from every available child in village A and from children in every other surveyed compound in village B within time constraints. Samples of manufacturer lots of materials used for blood collection were prescreened by CDC laboratories and determined to be free of lead. To prevent sample contamination, all blood collection supplies were kept in plastic gallon-size storage bags before sample collection. In addition, the venipuncture site was thoroughly cleaned with alcohol wipes before the specimen was obtained. One 1- to 3-mL blood sample was collected in a laboratory tube and analyzed for lead using a portable blood lead analyzer, LeadCare II® (Magellan Biosciences, Chelmsford, MA, USA) in an uncontaminated area away from the villages. This portable instrument can reliably determine BLLs from 3.3 to 65 µg/dL with an accuracy level of ± 3 µg/dL (Freeney and Zink 2007).

A second blood sample was collected from every third child and analyzed for lead, total mercury, and manganese by the Inorganic and Radiation Analytical Toxicology Laboratory at the CDC National Center for Environmental Health in Atlanta, Georgia. Inductively coupled plasma mass spectroscopy was used to analyze lead and total mercury. Detailed explanations of CDC blood lead and total blood mercury laboratory methods have been published elsewhere (Caldwell et al. 2009; Jones et al. 2007). Inductively coupled dynamic reaction cell plasma mass spectrometry was used for whole-blood manganese analysis as previously described (Jones et al. 2010). The limit of detection for blood lead is 0.25 µg/dL, total blood mercury 0.33 µg/L, and manganese 0.8 µg/L. Precision was evaluated by monitoring the replicate results of internal quality control (QC) materials. QC tests, included at the beginning and end of each analytical run, help ensure the accuracy and precision of the analysis process. The low-level QC should be in the low to normal range for blood levels in the U.S. population, and the high-level QC should be less than that found in the high-normal population range of the U.S. population. The population ranges are taken from the *Fourth National Report on Human Exposure to Environmental Chemicals* (CDC 2009). The low-level QC for mercury had an interday coefficient of variation (CV) of 15.1 at 0.516 µg/L and a CV of 2.3 at 5.857 µg/L. For lead, the low-level QC had an interday CV of 1.7 at 2.876 µg/dL and a CV of 1.2 at 12.754 µg/dL. For manganese, the low-level QC, with a mean of 7.983, has a CV of 4.8%, whereas the high-level QC with a mean of 14.929 has a CV of 6.7%. Accuracy for blood lead and mercury was verified by analyzing standard reference material from the National Institute of Standards and Technology (Gaithersburg, MD, USA) . Blood manganese accuracy was verified by participating in proficiency testing with the Wadsworth Center of New York Trace Elements in Whole Blood Program (Albany, NY, USA). The level of concern for blood lead is 10 µg/dL (CDC 2002). Although there is no blood level of concern for metallic mercury exposure, long-term effects, largely neurological, have been noted at total blood mercury levels < 1.0 µg/L (Agency for Toxic Substances and Disease Registry 1999). Investigators used the reference range of 7.7–12.1 µg/L from *Tietz Textbook of Clinical Chemistry* for blood manganese (Milne 1999). Although some heavy metals, such as mercury, are better evaluated using urine biomarkers, logistical constraints precluded collection of urine specimens.

*Environmental sampling.* Places where children ate or slept, which were identified by the eldest mother in each compound, were targeted for environmental sampling in each surveyed compound. Samples of soil were swept, placed in a plastic bag, and analyzed for lead content using a portable, hand-held X-ray fluorescence spectrometer (XRF) (Innov-XSystems, Woburn, MA, USA and Thermo-Scientific Niton, Billerica, MA, USA) in an uncontaminated area away from the villages. A certified industrial hygienist or environmental engineer also assessed each village and took XRF readings throughout the villages using U.S. Environmental Protection Agency (EPA) method 6200 to determine areas of high contamination (U.S. EPA 2007). Inside the villages, priority for XRF assessment and analysis was given to areas potentially affected by ore processing. The limit of detection for lead by XRF was approximately 40 ppm, whereas the upper reporting limit was 100,000 ppm. Samples of processed ore were also collected and analyzed for lead using XRF. After XRF analysis, a subset of soil and ore samples was sent to the U.S. Geological Survey (USGS) for further analysis using inductively coupled plasma mass spectroscopy to verify XRF field results and to perform mineralogy analysis to determine the chemical composition of the samples.

The U.S. EPA’s Lead: Renovation, Repair, and Painting Program (U.S. EPA 2008) defines a soil lead hazard as bare soil containing total lead ≥ 400 ppm in a child’s play area or 1,200 ppm in bare soil in other parts of a yard where a child lives (U.S. EPA 2008). U.S. EPA standards were used to categorize XRF soil lead results from surveyed compounds into three groups: ≤ 400 ppm, 401–1,200 ppm, and > 1,200 ppm. A limited number of water samples were collected by a certified industrial hygienist. Water was drawn up in a bucket, and the sampling bottle was immediately submerged to obtain the sample. Water samples were stabilized with 1 mL nitric acid. Unfortunately, no field blanks were taken. Water samples were sent to a commercial environmental testing laboratory in the United States and analyzed using U.S. EPA method 200.8 for lead, arsenic, and manganese. The U.S. EPA action level of 15 ppb for drinking water was the reference standard for water results (U.S. EPA 1991).

*Statistical analysis.* Data from the household survey, blood lead results, and environmental sample results were entered into Epi-Info version 3.5.1 (CDC, Atlanta, GA, USA). Statistical analyses were performed using SAS software (SAS Institute Inc., Cary, NC, USA). Univariate analyses, including calculation of daily and overall under age 5 mortality rates (U5MR) were performed. The daily U5MR was calculated by taking the number of deaths per 10,000 children per day for the 6-month period of December 2009—May 2010. This rate was compared with the United Nations High Commissioner for Refugees (UNHCR) threshold of > 2.0/10,000/day, which indicates deteriorating conditions in a refugee relief situation (UNHCR 2007). To calculate the overall U5MR, the number of deaths was divided by the number of children alive in the compounds in the 12-month period of May 2009–May 2010 and multiplied by 1,000. This was compared with the U5MR per 1,000 live births for this region of northwestern Nigeria (217 in 1,000 live births) as a way to crudely estimate excess mortality in the villages (NPC and ICF Macro 2009).

Bivariate and multivariate logistic regression was used to identify risk factors for child mortality from suspected lead poisoning. Using this outcome, compounds without any children < 5 years of age were excluded from bivariate and multivariate analyses. Risk factors were grouped into demographic, ore processing, and other environmental risk factors. Demographic risk factors included age, sex, and village of residence. Ore-processing risk factors included whether members of the compound or a child’s mother participated in any of the six ore-processing activities. Investigators focused on maternal risk factors because children < 5 years of age in this population typically spend the majority of time with their mothers in the compound. Environmental risk factors included proximity to ore-grinding activities, presence of ground material inside the compound, soil lead concentrations within the compound, primary water source, and history of animal death (as a proxy for environmental contamination) within the compound. The outcome of interest was death of a child. Multicollinearity, intervariable correlation, the Hosmer–Lemeshow goodness-of-fit assessment, and the effect of each risk factor on the outcome were considered. Variables from bivariate analysis with a *p*-value < 0.1 were tested in the model using backward selection. Variables with a *p*-value of < 0.05 remained in the final model.

## Results

*Survey results.* All 54 compounds identified in village A and 90% (65 of 72) of compounds in the central area of village B participated in the survey (*n* = 119). Responses from one compound in village A were excluded because data were incomplete. A total of 463 children < 5 years of age living in the sampled areas of the villages from May 2009 to May 2010 were included in this analysis. Data from all 118 surveyed compounds were used to calculate descriptive statistics regarding village demographics and ore-processing activities. To calculate bivariate and multivariate statistics, only data from the 110 surveyed compounds with children < 5 years of age were used.

The two villages were similar demographically (Table 1). An average of 4.2 children < 5 years of age lived in each compound. From May 2009 to May 2010, 25% (118 of 463) of children < 5 years of age in the surveyed compounds were reported to have died and 82% (97 of 118) were reported to have had convulsions before death. A history of convulsions was reported in 17% (58 of 345) of the surviving children. Sixty-two (53%) of 110 surveyed compounds with children < 5 years of age reported that from one to eight children < 5 years of age had died from May 2009 to May 2010. Eighty-two percent of deaths occurred from December 2009 to May 2010, making the daily U5MR 12.1 per 10,000 children age < 5 years/day for the period in the villages. The approximate overall U5MR for the 1-year period (May 2009–May 2010) was 255 per 1,000 live births.

**Table 1:** Demographics of participating family compounds by village.

Demographic	Village A (*n* = 53)*a*	Village B (*n* = 65)*a*	Both villages (*n* = 118)*a*
Mean no. of married men per compound		3.1		2.1		2.6
Mean no. of mothers per compound		3.7		3.0		3.4
Mean no. of children < 5 years of age per compound		4.8		3.1		4.2
Total no. of children < 5 years of age in participating compounds, as of May 2009		259		204		463
No. of children < 5 years of age living at time of survey (%)		181 (70)		164 (80)		345 (75)
No. of children < 5 years of age who had died within previous 12 months before survey (%)		78 (30)		40 (20)		118 (25)
No. of compounds with ≥ 1 pregnant women (%)		26 (49)		24 (37)		50 (42)
**a**Number of compounds.						

Overall, 71% (84 of 118) of compounds reported processing gold ore within their compound (Table 2). Compounds participating in ore processing averaged four different processing activities. Ore drying was performed more frequently in compounds in village A than in village B (69% vs. 49%, *p* = 0.002). Only 25 compounds reported grinding activities within their compound (Table 2), but 46% (54 of 118) reported grinding activities nearby, and 27% (32 of 118) of compounds reported having ground ore present in their compound. Fifty-six of the 84 (67%) compounds processing ore started doing so in the 12 months preceding the investigation (May 2009–May 2010); however, some compounds (33%) reported processing for as long as 18 years. Compounds in village B were more likely to use a well inside their compound than those in village A, who were more likely to use a community well as their main water source (94% vs. 60%, *p* < 0.001).

**Table 2:** Participation in ore processing within family compounds by village [no. (%)].

Activity	Village A (*n* = 53)*^a^*	Village B (*n* = 65)*^a^*	Both villages (*n* = 118)*^a^*
Participate in ≥ 1 activities		42 (79)		42 (65)		84 (71)
Rock breaking		29 (55)		39 (60)		68 (58)
Rock grinding		10 (19)		15 (23)		25 (21)
Rock washing		27 (51)		27 (42)		54 (46)
Rock drying		33 (62)*		29 (45)*		62 (53)
Rock separating		22 (42)		30 (46)		52 (44)
Rock melting		22 (42)		32 (49)		54 (46)
Mean number of activities		3.4		4.1		3.8
**a**Number of compounds. *p < 0.05.						

*Blood sampling.* Venous blood lead samples were obtained from 59% (204 of 345) of children < 5 years of age. All blood samples indicated lead poisoning (BLL ≥ 10 µg/dL). For 97% (198 of 204) of children, BLLs were ≥ 45 µg/dL, the CDC-recommended threshold for initiating chelation therapy (CDC 2002). Eighty-five percent (173 of 204) of blood samples exceeded the maximum detection limit of the LeadCare II instrument (65 µg/dL).

Eighty-six blood samples were sent to CDC laboratories for blood lead, mercury, and manganese testing (Table 3). The mean blood lead concentration for children in village A was 153.3 µg/dL (range 55.9–331.0 µg/dL) and 107.5 µg/dL (range 36.5–445.0 µg/dL) for children in village B. The mean total blood mercury level for children in village A was 2.4 µg/L (range 0.3–6.6 µg/L) and 1.4 µg/L (range 0.4–6.5 µg/L) in village B. For comparison, the geometric mean value for total blood mercury reported for children 1–5 years of age in the *Fourth National Report on Human Exposure to Environmental Chemicals* was 0.33 µg/L [95% confidence interval (CI): 0.29, 0.37], with the 95th percentile measuring 1.8 µg/L (95% CI: 1.3, 2.5) (CDC 2009). Mean blood manganese levels were 14.0 (range 4.0–39.0 µg/L) and 20.9 (range 4.9–40.8 µg/L) in villages A and B, respectively; 66% (57/86) of samples were above the reference range of 7.7–12.1 µg/L for manganese.

**Table 3:** Blood lead, manganese, and mercury concentrations by village.

Lead (µg/dL)					Manganese (µg/L)					Mercury (µg/L)				
Village	*n*	Mean	Median	Range	Mean	Median	Range	Mean	Median	Range
Village A		44		153.3		143.8		55.9–331		14.0		11.6		4.0–39.0		2.4		2.1		0.3–6.6
Village B		42		107.5		85.7		36.5–445		20.9		20.1		4.9–40.8		1.4		0.99		0.4–6.5
Reference range for lead is < 10 µg/dL and for manganese is 7.7–12.1 µg/L. Health effects of mercury can be seen at > 1 µg/L.																				

*Environmental sampling.* Soil obtained from 116 of 118 compounds was analyzed in the field. Soil lead concentrations ranged from 45 ppm to > 100,000 ppm; 85% of samples exceeded the U.S. EPA threshold of 400 ppm. The average soil lead concentration from household samples analyzed by XRF was 7,959 ppm (range 421 to > 100,000 ppm) in village A and 3,298 ppm (range 45 to > 100,000 ppm) in village B. Four water samples were collected and sent to a commercial environmental testing laboratory in the United States for analysis (Bureau Veritas Laboratory, Novi, MI). Community wells in villages A and B had 520 ppb and 1,300 ppb of lead, respectively, exceeding the U.S. EPA action level of 15 ppb lead in drinking water (U.S. EPA 1991). Two convenience water samples, one from a pond and one from a private well in village B, were also tested and had lead concentrations of 250 and 37 ppb lead, respectively. The private well was selected because no ore-processing impact was suspected. The pond was selected because it had been used for sluicing gold ore, and village livestock were observed using this water source. The lead concentrations of five ore samples from various stages of ore processing measured at USGS laboratories were 331, 9,150, 13,700, 112,000, and 175,000 ppm. This confirmed that some gold ore processed by the villagers was rich in lead content.

*Bivariate and multivariate analyses*. Table 4 summarizes the results of bivariate analyses. Children who died were more likely than children who survived to have had a mother who participated in ore-processing activities [odds ratio (OR) = 1.9; 95% CI: 1.2, 2.9]. Having a mother who participated in grinding, washing, drying, separating, or melting activities was significantly associated with a child’s death (*p* < 0.05). Children who died were more likely than children who survived to have ground ore present in the compound (OR = 2.0; 95% CI: 1.3, 3.2). Proximity to village grinding activities was not significantly associated with mortality in bivariate analysis.

**Table 4:** Potential risk factors for child mortality.

Outcome		
Potential risk factor	Deceased	Alive	Crude OR (95% CI)	*p*-Value
Maternal activities								
Breaks ore						1.5 (0.96, 2.2)		0.08
Yes		56		132				
No		62		213				
Grinds ore						2.6 (1.4, 5.0)		0.002
Yes		20		25				
No		98		320				
Washes ore						2.6 (1.4, 4.7)		0.002
Yes		22		28				
No		96		317				
Dries ore						2.9 (1.8, 4.8)		< 0.0001
Yes		38		48				
No		80		297				
Separates ore						1.7 (1.0, 3.1)		0.05
Yes		23		42				
No		95		303				
Other environmental risk factors								
Mother performs ≥ 1 ore-processing activity		74		163		1.9 (1.2, 2.9)		0.0037
Mother performs 0 ore-processing activities		44		182				
Soil lead level in compound (ppm)								
< 400		8		53		Reference		Reference
400–1,200		20		111		1.2 (0.5, 2.9)		0.7
> 1,200		90		178		3.4 (1.5, 7.4)		0.0026
Dried ore in compound						2.0 (1.3, 3.2)		0.0041
Yes		46		82				
No		72		263				
Main water source						3.4 (2.0, 5.7)		< 0.0001
Community well		35		38				
Private well		83		307				
History of animal death in compound						2.4 (1.5, 3.8)		0.0003
Animal death		89		195				
No animal death		29		150				
Demographic risk factors								
Age								
Birth–24 months		88		181		2.6 (1.6, 4.2)		< 0.0001
25–49 months		30		161				
Village of residence						1.8 (1.1, 2.7)		0.01
Village A		78		181				
Village B		40		164				


Environmental and demographic variables tested in the logistic regression model are presented in Table 4. Although we collected data regarding whether any household member performed ore-processing activities as well as if the child’s mother performed ore-processing activities, the two responses were not mutually exclusive and were highly correlated in bivariate analysis. Because previous studies have found that a child’s BLL is most related to the mother’s activities and BLL (CDC 2002), we evaluated only risk factors related to the mother’s activities in the logistic regression model. Child mortality was significantly related to five risk factors in the final model: age of the child, whether the mother performed ore breaking or washing activities, whether a community well was used as a primary water source, and the soil lead level of the child’s compound (Table 5). No potential interaction terms were included in the final model, as they did not improve explanatory power of the model.

**Table 5:** Significant risk factors retained in final model.

Risk factors	Crude OR (95% CI)	*p*-Value	Adjusted OR (95% CI)
Maternal activities						
Breaks ore		1.5 (0.96, 2.2)		0.08		1.8 (1.1, 3.0)
Washes ore		2.6 (1.4, 4.7)		0.002		3.4 (1.7, 6.7)
Other environmental risk factors						
Lead level in compound (ppm)						
< 400		Ref		Ref		Ref
400–1,200		1.2 (0.5, 2.9)		0.7		1.5 (0.6, 3.8)
> 1,200		3.4 (1.5, 7.4)		0.0026		3.6 (1.5, 8.5)
Main water source		3.4 (2.0, 5.7)		< 0.0001		3.7 (2.0, 6.8)
Demographic risk factors						
Age (birth–24 months vs. 25–49 months)		2.6 (1.6, 4.2)		< 0.0001		2.6 (1.6, 4.4)

After controlling for the maternal ore-processing activities of breaking and washing and the environmental factors including water source and soil lead level within the compound, we found that children ≤ 24 months of age were 2.7 times as likely to have died as children 25–49 months of age (95% CI: 1.6, 4.4). In addition, children living in a compound with a soil lead level of > 1,200 ppm were 3.6 times as likely to have died as children in a compound with a soil level of < 400 ppm (95% CI: 1.5, 8.5), and children whose primary water source was a community well outside their compound were 3.7 times as likely to have died as children whose primary source was a well inside the compound (95% CI: 2.0, 6.8).

## Discussion

The United Nations High Commission on Refugees (UNHCR) considers a daily U5MR of > 2.0/10,000/day a key indicator that conditions in a refugee situation are deteriorating (UNHCR 2007). The daily U5MR of this outbreak was more than six times the UNHCR benchmark and well above the overall U5MR in the region (217 per 1,000) and in the nation (157 per 1,000). Children ≤ 24 months of age had 2.6 times the odds of death compared with older children, consistent with previous research that indicates that younger children are at greater risk for lead poisoning.

This investigation was subject to at least four limitations. First, postmortem evaluations and blood lead samples were not available for deceased children. Thus, not all of these deaths can be reliably attributed to lead poisoning, and BLLs in children who died cannot be compared with those who lived. Second, we were unable to determine whether other unmeasured cultural or nutritional factors exist that may explain some of the high mortality rates. Third, the survey and environmental sampling were conducted by local staff with limited training, and no field blanks were obtained to validate sampling methods. Variation could have occurred in the way survey questions were asked or how environmental samples were collected. Finally, we were able to determine only an approximate U5MR for the villages before the outbreak based on regional data.

Despite these limitations, it is reasonable and prudent to conclude that most of the recent childhood deaths in these villages were caused by acute lead poisoning and to take steps to stop the exposure. This conclusion is based on high environmental lead concentrations, the percentage of children with elevated BLLs (97% > 45 µg/dL), the high incidence of convulsions before death (82%), and the significant reduction in mortality once treatment for lead poisoning was initiated and children were removed from the source of contamination. Although mortality was reduced, it will take some time to fully characterize the extent of neurological damage that has occurred in these children. In this investigation, 85% of surveyed compounds had soil lead levels > 400 ppm, and some had levels 250 times the U.S. EPA threshold. Most compounds in this rural, impoverished setting were constructed of dirt walls and floors. Children came in direct contact with large amounts of soil through daily activities, and this provides a constant and direct route of exposure for children. We also demonstrated a relationship of increasing exposure related to increasing odds of dying, because children whose compounds contained ≤ 1,200 ppm lead were less likely to die than children whose compounds contained > 1,200 ppm lead. Moreover, environmental contamination may not be limited to soil, and contamination of water sources is also possible. In our multivariate model, the odds of death were 3.7 times greater for children who had a community well as their main source of water compared with children whose main water source was a private well in their compound. Four water samples, including two from community wells, had lead concentrations that greatly exceeded the U.S. EPA action level for drinking water of 15 ppb.

Most of the compounds that engaged in ore-processing activities (67%) began them in the 12 months preceding this investigation, when child mortality also increased. Although not captured in our survey, anecdotal evidence indicates that the increase in child mortality was related to the increased use of flour-grinding machines located within the villages to grind ore. Before November 2009, at least one grinder was reported by villagers to be in each of the surveyed villages, but after November 2009, villagers reported that there were up to 10 grinding machines in each village. The increase in the number of grinding machines would likely amplify the amount of ground ore around the village and in individual compounds, contributing to widespread contamination. The increase in lead contamination and continued use of mercury also warrant investigation into the health effects from multiple metals in children and adults. Further research on multiple metal exposures in this population is needed.

During and after this investigation, accounts of similar ore-processing activities and increased rates of child mortality were reported in other nearby villages. Characterizing the full extent of the outbreak remains an urgent and ongoing matter. As more affected villages are identified, medical treatment and environmental remediation will require trained personnel and increased funding. Initiating medical treatment in the absence of environmental remediation is not only ineffective in reducing children’s lead exposure and body burden, but also may cause more harm than good (Chisholm 1992). CDC recommendations for follow-up and rehabilitation of lead-poisoned children in the United States prioritize control or elimination of sources of exposure as the most essential intervention, and great progress has been made to reduce lead exposure (CDC 2002). In these villages in Nigeria, contamination of water systems, crops, and animals, as well as the risk of recurrent contamination from villages who temporarily cease and then resume ore processing, remain enormous issues to be addressed. Community-based education campaigns are ongoing. Messages include the need for blood lead testing and environmental remediation and the dangers of ore processing, particularly within the confines of the village.

Beginning in June 2010, chelation therapy was provided free of charge in hospitals supported by MSF and Nigerian staff for all children < 5 years of age in the surveyed villages. Hundreds of children have undergone chelation therapy, and unpublished data from MSF clinics in these villages indicate that child mortality has been reduced from ~ 43% immediately before the investigation to < 1% postinvestigation. Each compound in the villages has been remediated to decrease the risk for lead poisoning. Environmental remediation efforts spearheaded by TerraGraphics (Moscow, ID, USA) and the Blacksmith Institute (New York, NY, USA), with the support of Nigerian state and local officials, began in early June 2010. TerraGraphics/Blacksmith Institute provided technical assistance and training for the remediation, and the manual labor was largely performed by Nigerian state, local, and village members. Remediation included removing contaminated surface soil and replacing it with clean uncontaminated soil and disposing of contaminated soil in secure landfills. Remediation drastically brought down soil lead levels inside compounds and reduced exposure of the children to lead. Remediation efforts also included education messages emphasizing the importance of prohibiting ore-processing activities and ore-processing materials inside the compound. However, postremediation follow-up is necessary to determine the long-term efficacy of the remediation.

Unfortunately, the type of artisanal and small-scale mining (ASM) demonstrated in this article is occurring on a global scale. According to the Global Mercury Project, an initiative of the United Nations, ASM occurs in > 55 countries, and 10–15 million miners work globally, primarily in Africa, Asia, and South America. An estimated 100 million people globally rely directly or indirectly on ASM for their livelihood (Spiegal and Veiga 2007). For many individuals, participation in mining is driven by poverty and a lack of economic opportunities, especially in rural communities, as seen in this outbreak. ASM is the source of 20–30% of the world’s gold (Spiegal and Veiga 2007). The price of gold continues to rise on the global market, and during the last decade gold has increased 360% (Swenson et al. 2011).

ASM has long been recognized as having negative health and environmental impacts. Mercury exposure in mining communities has been well established in the literature in places such as Brazil, Indonesia, Zimbabwe, and Ghana (Global Mercury Project 2006; Kwaansa-Ansah et al. 2010), but much less is known about other toxic chemicals, such as lead. Findings from this investigation should be included into existing global initiatives to address other heavy metals in addition to mercury.

## Conclusion

In this article we describe an outbreak of childhood lead poisoning related to artisanal mining that resulted in child mortality rates unprecedented in modern times. To our knowledge, this is the first documentation of an outbreak of childhood lead poisoning associated with artisanal gold mining. Twenty-five percent of children < 5 years of age in the two villages died from May 2009 to May 2010, and 97% of surviving children had BLLs that required chelation therapy. Extensive environmental contamination was found in both of the villages and inside individual family compounds. The response to this outbreak required extensive coordination with federal and state ministries, international nongovernmental organizations, international organizations, and U.S. government agencies.

The need for technological assistance and transfer in countries with developing economies and health care infrastructure is critical. In resource-limited areas such as northern Nigeria, use of natural resources is an economic necessity. However, mining has exposed the communities to high lead concentrations, with devastating effects on the population. Affected children in these villages may suffer long-term consequences such as intellectual deficits and blindness. Safer mining practices, including moving the processing away from village, techniques to reduce dust generation, and basic hygiene could prevent lead exposure and subsequent lead poisoning in persons participating in ore-processing activities and their families.
